# TraumaFlow—development of a workflow-based clinical decision support system for the management of severe trauma cases

**DOI:** 10.1007/s11548-024-03191-2

**Published:** 2024-05-30

**Authors:** Juliane Neumann, Christoph Vogel, Lisa Kießling, Gunther Hempel, Christian Kleber, Georg Osterhoff, Thomas Neumuth

**Affiliations:** 1 Innovation Center Computer-Assisted Surgery (ICCAS), Leipzig University, Leipzig, Germany; 2 Department of Orthopedics, Trauma and Plastic Surgery, University Hospital Leipzig, Leipzig, Germany; 3 Department of Anesthesiology and Intensive Care, University Hospital Leipzig, Leipzig, Germany

**Keywords:** Workflow management, Clinical decision support, BPMN 2.0, Polytrauma, Resuscitation room

## Abstract

**Purpose:**

The treatment of severely injured patients in the resuscitation room of an emergency department requires numerous critical decisions, often under immense time pressure, which places very high demands on the facility and the interdisciplinary team. Computer-based cognitive aids are a valuable tool, especially in education and training of medical professionals. For the management of polytrauma cases, TraumaFlow, a workflow management-based clinical decision support system, was developed. The system supports the registration and coordination of activities in the resuscitation room and actively recommends diagnosis and treatment actions.

**Methods:**

Based on medical guidelines, a resuscitation room algorithm was developed according to the cABCDE scheme. The algorithm was then modeled using the process description language BPMN 2.0 and implemented in a workflow management system. In addition, a web-based user interface that provides assistance functions was developed. An evaluation study was conducted with 11 final-year medical students and three residents to assess the applicability of TraumaFlow in a case-based training scenario.

**Results:**

TraumaFlow significantly improved guideline-based decision-making, provided more complete therapy, and reduced treatment errors. The system was shown to be beneficial not only for the education of low- and medium-experienced users but also for the training of highly experienced physicians. 92% of the participants felt more confident with computer-aided decision support and considered TraumaFlow useful for the training of polytrauma treatment. In addition, 62% acknowledged a higher training effect.

**Conclusion:**

TraumaFlow enables real-time decision support for the treatment of polytrauma patients. It improves guideline-based decision-making in complex and critical situations and reduces treatment errors. Supporting functions, such as the automatic treatment documentation and the calculation of medical scores, enable the trauma team to focus on the primary task. TraumaFlow was developed to support the training of medical students and experienced professionals. Each training session is documented and can be objectively and qualitatively evaluated.

## Introduction

Trauma is one of the leading causes of death for young and middle-aged patients worldwide. The injuries, resulting mostly from accidents, falls, burns, and violence, contribute to approximately 4.4 million deaths each year [1]. Trauma patients often have life-threatening injuries to multiple body parts or organ systems. The treatment is highly complex, time-sensitive, and requires a multidisciplinary team and special medical resources [2]. A systematic assessment of the injury patterns as well as a structured therapy with prompt decisions are necessary to increase the likelihood of survival [3]. Thereby, the trauma team has to make highly critical decisions based on numerous parameters and patient attributes in a short time [4, 5]. Different tasks take place simultaneously or in close succession, which places very high demands on the resources of the facility and the interdisciplinary team [6].

The introduction of standardized treatment protocols and training courses (e.g. Advanced Trauma Life Support (ATLS) [7], European Trauma Cours (ETC) [8]), the establishment of specialized trauma centers, and trauma data registers improved the quality of care and reduced patient mortality in the last decade [9, 10]. However, diagnostic and treatment errors continue to occur in about 10% of the cases in the treatment of acute trauma [4, 11]. Human failure is the most important origin of errors, mainly caused by wrong treatment, delayed treatment, and the omission of an essential therapy [11]. The reported rates of preventable trauma deaths due to erroneous treatment range from about 2–22% [12–14]. Compliance with standardized treatment protocols ranges from only about 40–80% [4, 15, and 16]. The study results imply that improved compliance with standardized protocols may lead to a better treatment outcome [15, 16]. These treatment protocols are evidence- and consensus-based guidelines on best practices for diagnosis, management, and therapy, which tend to be neither patient-specific nor contain explicit decision-making statements. Computer-aided decision support systems (CDSS) can implement those guidelines and assist in the standard-compliant and patient-specific management of polytrauma care. Thereby, objective and accurate assistance for decision-making as well as improved resource allocation can be provided [4].

In this study, TraumaFlow a workflow-based CDSS for the management of severe trauma cases, was developed. The main aim of TraumaFlow is to support the education and training of trauma team members in guideline-based critical decision-making, with the ultimate goal of reducing errors during polytrauma treatment. For this purpose, the system provides real-time decision support with recommendations for action, patient-specific treatment management, and several assistance functionalities (e.g., process documentation and medical score calculation). This study aimed to technically validate the CDSS, which was assessed in different simulated polytrauma scenarios during training sessions. Thereby, the effect of the assistance on decision-making and guideline adherence, as well as the human-system interaction, were analyzed.

### State-of-the-art

CDSS are software tools representing algorithms and providing decision support either for diagnosis, treatment, or both. The more sophisticated interpretation systems collect patient parameters as well as diagnostic and treatment measures and suggest further therapy steps based on calculated probabilities or expert-defined rules and guidelines [17]. A complete overview of paper-based and electronic CDSS for emergency situations is provided by Greig et al. [18].

In the domain of polytrauma care, the necessity for CDSS was recognized at an early stage. TraumaAID, which was developed in the early 1990s, used decision rules and logical deduction to create a treatment plan for severely injured patients [19]. In an evaluation study, TraumaAID was able to reduce preventable mistakes by 98% [20]. In contrast to TraumaFlow, the system used a fixed treatment plan and cannot provide real-time and context-sensitive information about the individual patient. Another CDSS is TraumaSCAN, which was specifically developed for the assessment of penetrating trauma in intrathoracic and intraabdominal injuries [21]. Fitzgerald et al. presented a CDSS, which is an algorithm-based display of a computer-prompted algorithm for real-time use on patients with major trauma [4]. The system checks possible diagnoses and recommends appropriate treatment options without being context-sensitive to the actual patient.

To the best of our knowledge, TraumaFlow is the first workflow-based resuscitation room CDSS to provide active, real-time, and patient-individual recommendations for the treatment of polytrauma patients based on standardized polytrauma treatment protocols and guidelines. In addition, context-sensitive assistance functionalities and treatment documentation were realized.

## Material and methods

### Development of the trauma treatment process models

The main task of trauma treatment is to restore and maintain the patient's vital signs and circulation. Therefore, diagnostic measures and immediate life-saving interventions are performed in a resuscitation room (e.g., resuscitation, infusions, or blood transfusions). All these measures occur concurrently or in rapid succession, imposing significant demands on both the resources of the facilities and the interdisciplinary team [6]. Therefore, certified trauma centers in Germany must fulfill various requirements to ensure high quality and safety in trauma care [22]. Mainly, the centers need to (1) provide a trained trauma team (minimally consisting of a trauma leader, an orthopedic surgeon, an anesthetist, and one emergency or anesthesia nurse) and appropriate infrastructure (e.g., resuscitation room, medical equipment), (2) adhere to standardized treatment protocols based on evidence-based guidelines [22], (3) participate regularly in mandatory special training programs (e.g., ATLS, ETC [7]) and (4) participate in internal and external quality assurance measures via trauma data registers (e.g. DGU Trauma Register [23]).

The trauma leader coordinates the treatment process, performs priority-based decision-making, and determines the diagnostic and therapeutic measures according to the cABCDE approach. The approach is used for a structured initial examination of a patient and starts with the most life-threatening condition. Thereby, the patient is treated in a structured way and is repeatedly assessed from the perspectives of: c—critical bleeding (treatment of immediate life-threatening bleeding), A—Airway (secure or establish airway), B—Breathing (ensure adequate gas exchange), C—Circulation (ensure circulation and adequate tissue perfusion), D—Disability (recognize neurological deficits), and E—Environment (complete full body examination). During this treatment process, the evidence- and consensus-based German “S3-Guideline on the Treatment of Patients with Severe/Multiple Injuries” [22] provides an aid to decision-making in specific situations. In addition, the trauma team members are trained according to the ATLS/ETC protocol, which teaches standardized, priority-oriented resuscitation room management.

For TraumaFlow a detailed resuscitation room algorithm was developed according to the cABCDE approach based on the S3-Guideline [22]. The semiformal algorithm was created by a medical expert and evaluated in a consensus-based approach by senior trauma physicians. The algorithm covers decisions, diagnosis, and treatment options for the cABCDE evaluation as well as for circulatory shock and resuscitation situations. Then, the algorithm was modeled formally with the process modeling standard BPMN 2.0 [24] and technically enriched with process parameters for execution with a workflow management engine. Additionally, complex decisions were modeled using the DMN standard [25] (e.g., calculation of the Glasgow Coma Scale, tranexamic acid admission). In this way, numerous decision models and three workflow models were created for the workflow execution during polytrauma treatment (cABCDE main model, circulatory shock subprocess model, and resuscitation subprocess model).

### Development of the workflow management system TraumaFlow

The workflow models were implemented in the workflow management system (WfMS) *Flowable* [26]. Flowable offers an Open Source version, which comes with a web-based User Interface (UI) application, a modeling component for BPMN, DMN, and CMMN models, and a workflow engine management component. The WfMS enables the efficient execution of parallel tasks and human-system interaction via user forms. The front-end of Flowable was adapted for use in the resuscitation room by adding additional features (e.g., color-based prioritization of tasks, process- and resuscitation timer).

### Evaluation study

**Fig. 1:**
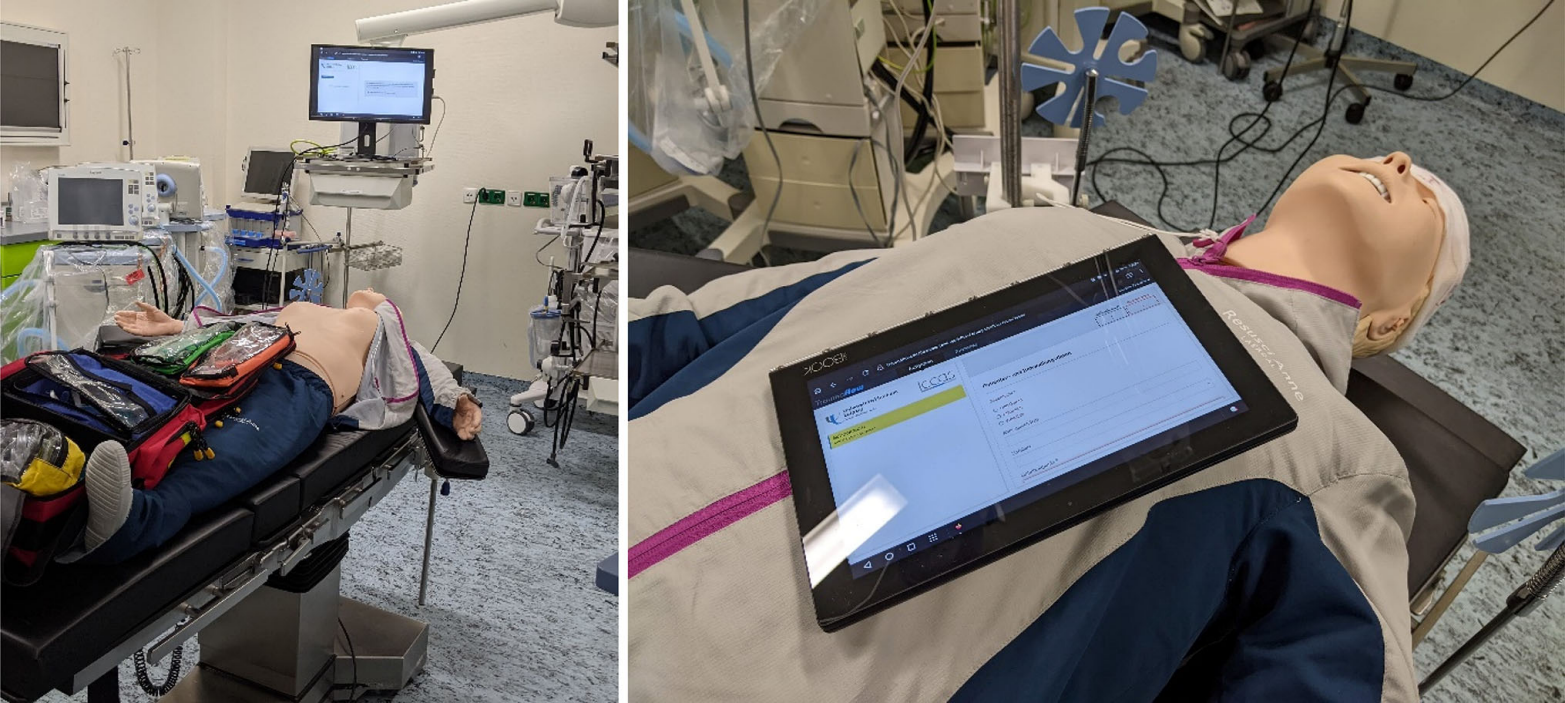
Polytrauma management training with a simulation mannequin (left) and TraumaFlow tablet computer (right)

An evaluation study was conducted to validate TraumaFlow technically and clinically in simulated polytrauma scenarios. On the one hand, the goal was to investigate the effect of computer-assisted decision support on guideline adherence, completeness of treatment, and errors, and on the other hand, to examine the system behavior and the human-system interaction. For this purpose, TraumaFlow was tested in an intervention room of a level-1 trauma center (Fig. 1, left) with 11 final-year medical students and 3 residents with more than 5 years of experience in the management of polytrauma patients. Of the 11 students, eight had experienced less than five polytrauma cases (low experience) and three had experienced 6–10 cases (medium experience). All participants were familiar with the ATLS/ETC principles, the cABCDE scheme, and the polytrauma guideline. The study participants were asked to act as the trauma leader, who coordinates the treatment process with two actors, representing a physician and an emergency nurse. Two scenarios were simulated for all participants. The first training session was conducted without computer-assisted support, and in the second session, TraumaFlow was used, which was accessed via tablet computer (Fig. 1, right). The participants were not familiar with TraumaFlow and received a short introduction to the system at the beginning of the scenario.

In case A, a male victim of a violent crime was injured with a blunt weapon. He was presented with a pneumothorax, a relevant bleeding scalp wound, and a splenic injury with a resulting class-III-hemorrhagic shock. In case B, a man fell from a 6 m-high scaffold, resulting in a severe traumatic brain injury with intracranial hemorrhage, an unstable pelvic injury, free fluid in the abdomen, and a class-III-hemorrhagic shock. The scenarios were altered to be performed with and without computer-assisted support and assessed by a certified trauma simulation instructor. When TraumaFlow was used in the scenario, the system logged the decisions made by the participants and the recommended actions. In addition, the system usage and scenarios without decision support were logged manually by a trained observer.

#### Evaluation of adherence to guidelines and treatment errors

Guideline adherence and completeness of care were rated, considering if the patient's status was assessed correctly and whether treatment measures were conducted appropriately. A score of up to 12 points could be achieved by gaining one point for completing an essential task successfully. A more detailed description of the clinical evaluation of TraumaFlow has been published previously [27].

#### Evaluation of the system behavior

The behavior of TraumaFlow was evaluated by recording usage errors of the participants (e.g., wrong parameter input) and system errors (e.g., wrong recommendation, unexpected behavior) during the scenario execution. In addition, the treatment time was documented for each scenario.

#### Evaluation of human-system-interaction

The human-system interaction was assessed with a user questionnaire, which was answered by the participants after the completion of both scenarios. The participants answered questions about the usability of the system by using the UEQ questionnaire [28] and completed seven Likert scale questions about their experience with TraumaFlow.

## Results

### Trauma treatment process models

The modeled cABCDE trauma treatment process starts with the patient arriving in the resuscitation room and the handover between emergency services and the trauma leader. Initial information about the patient (e.g., gender, accident type, time of accident) must be entered into the system. If necessary, the resuscitation and circulatory shock subprocess can be started directly and at any time during the process via a BPMN non-interrupting call activity, which starts the subprocess parallel to the main process. The main process inherits the variables automatically, and the subprocess can return the documented treatment variables to the main process.

**Fig. 2:**
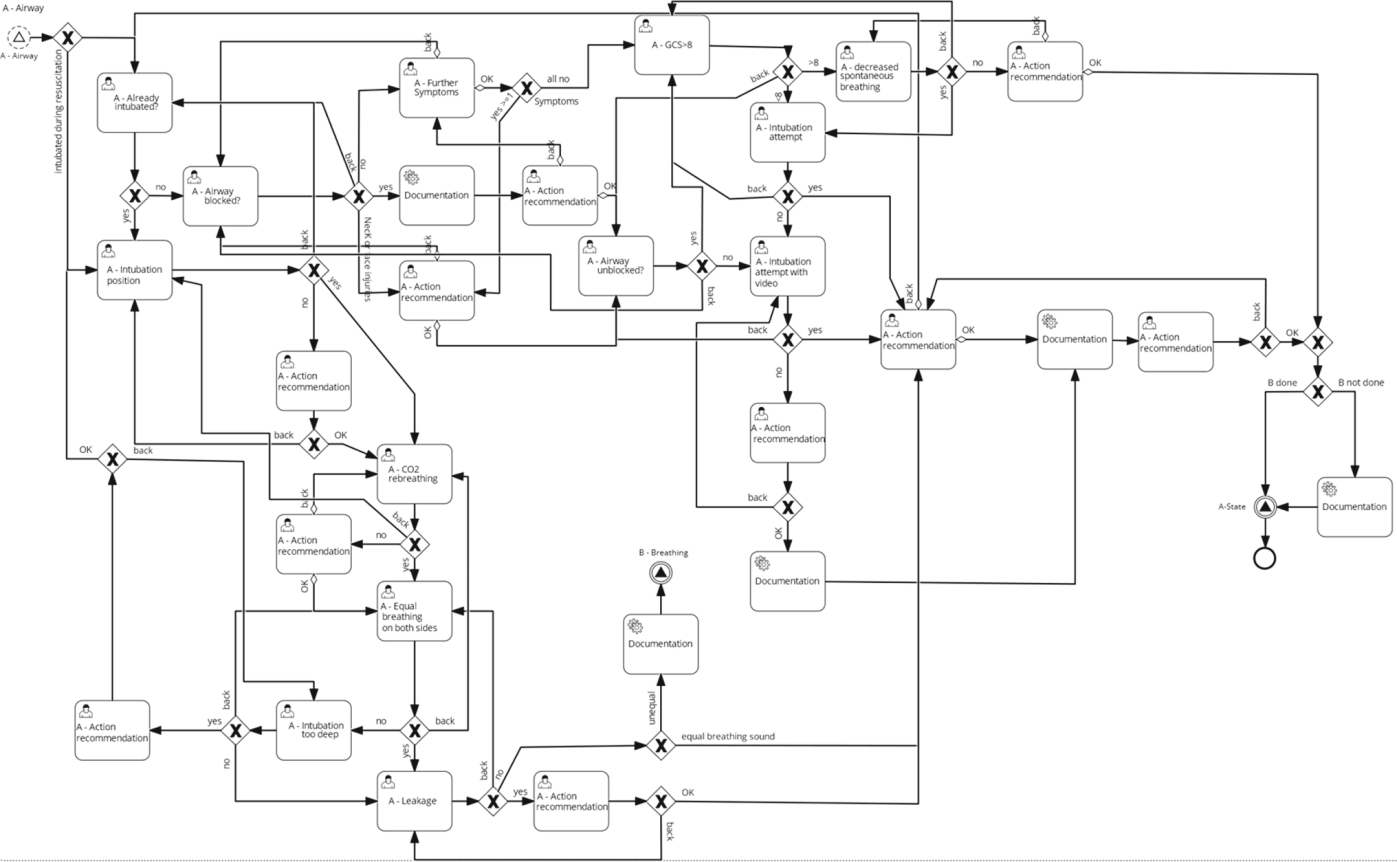
Example subprocess—Airway assessment in TraumaFlow modeled with BPMN

The main process is divided into grouped subprocesses, which are called via BPMN signal events and separate the individual treatment stages of the cABCDE approach. For example, the “A-Airway process” is shown in Fig. 2. For user interaction, the BPMN user task element is used, which creates an editable form during workflow execution. In this way, data can be submitted to the system, or a selection between different recommendations can be made. If a decision is made, an XOR gateway is used whenever only one subsequent sequence flow is selected. AND gateways are used to start parallel processes. For complex decisions and calculations, DMN tables are integrated into the process model via BPMN decision tasks. Also, external Java Code files (e.g., for protocol generation) are executed and triggered via BPMN service tasks.

In this way, the patient is evaluated by going through the cABCDE stages. At the end of each stage, the user is asked to reevaluate the finished stages (e.g., repeat B-stage when the breathing deteriorates). After a successful initial treatment in the resuscitation room, the patient is transferred either to the operating room or to the intensive care unit, and the process can be finished in TraumaFlow. Afterward, it is possible to generate treatment documentation.

### Workflow management system

**Fig. 3:**
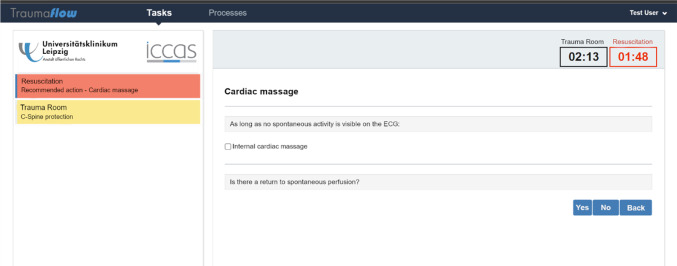
TraumaFlow user interface

In Fig. 3, the TraumaFlow user interface is shown, which is the visualized workflow instantiation of the modeled treatment process. On the left side, the currently active tasks are color-coded by priority. On the right side, detailed information about the current task and decision options is displayed. For every task, a ‘Back-Button’ was modeled to enable a return to the previous task. TraumaFlow provides additional features, such as a timer for the main treatment and the resuscitation process. During treatment, different medical scores (e.g., Injury Severity Score) can be calculated in real-time to improve decision-making. All the system-recommended actions can be traced through the underlying process model for explainability, which is also accessible during runtime. In addition, the WfMS logs every executed task and decision made to the second. Based on the system logs, TraumaFlow enables detailed documentation of the training session. Hence, a prefilled hospital internal handover protocol, the trauma protocol of the DGU trauma register, and a detailed training protocol are generated at the end of every process execution.

### Evaluation study

#### Adherence to guidelines and treatment errors

**Fig. 4:**
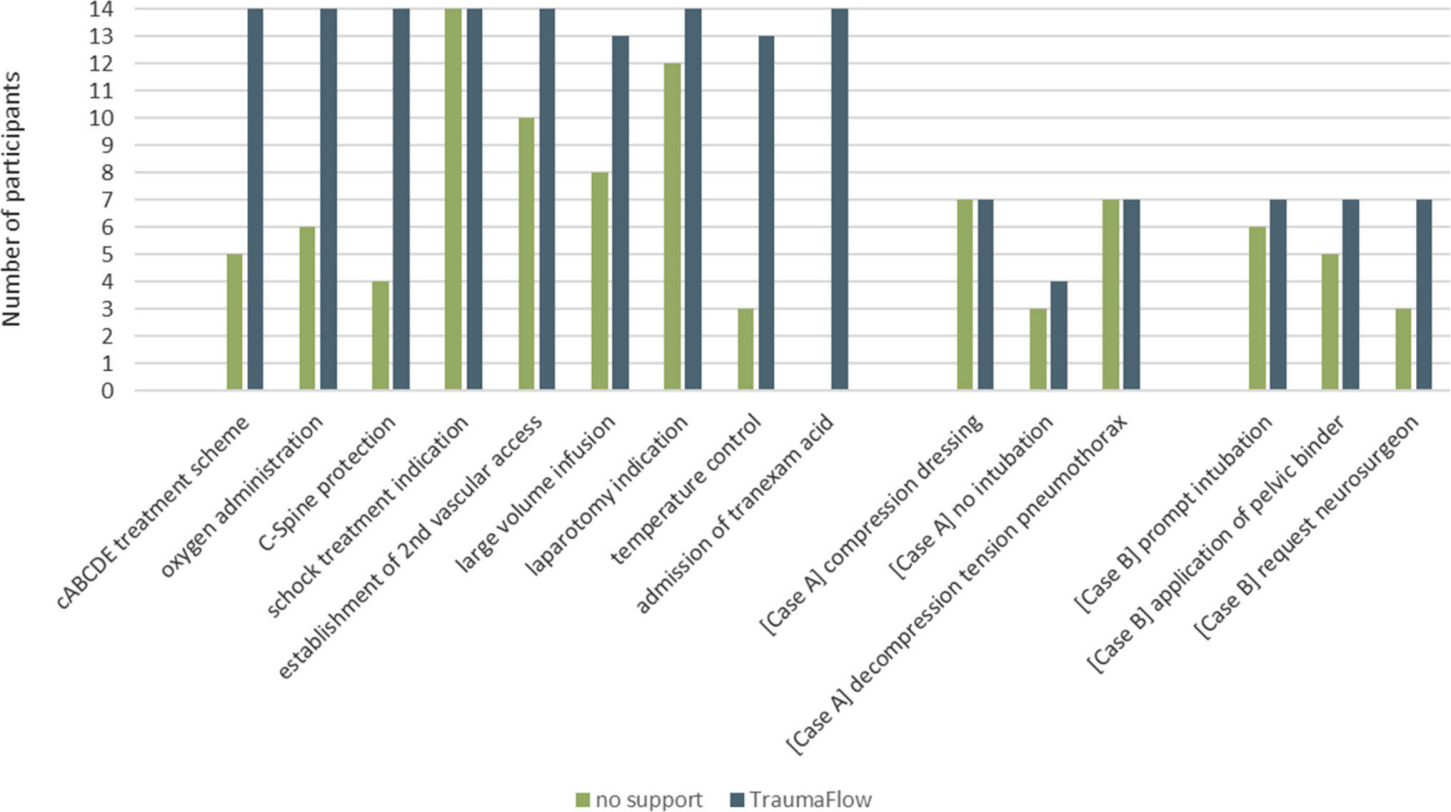
Essential treatment steps performed by 14 participants. Thereof, nine steps are generic (max. number of participants is 14) and three are scenario-specific (max. number is seven due to altered scenario execution)

To evaluate the guideline adherence, every scenario execution was rated by an experienced trauma instructor with up to 12 points for correctly recognized and applied treatment. In Fig. 4, the number of participants that perform essential treatment steps is shown for scenarios with and without TraumaFlow. For example, of all 14 participants, only five performed trauma treatment following the cABCDE scheme without decision support. In addition, tranexamic acid was not given once. With the use of TraumaFlow, all participants followed the cABCDE approach and applied tranexamic acid as proposed in the S3-Guideline.

**Fig. 5:**
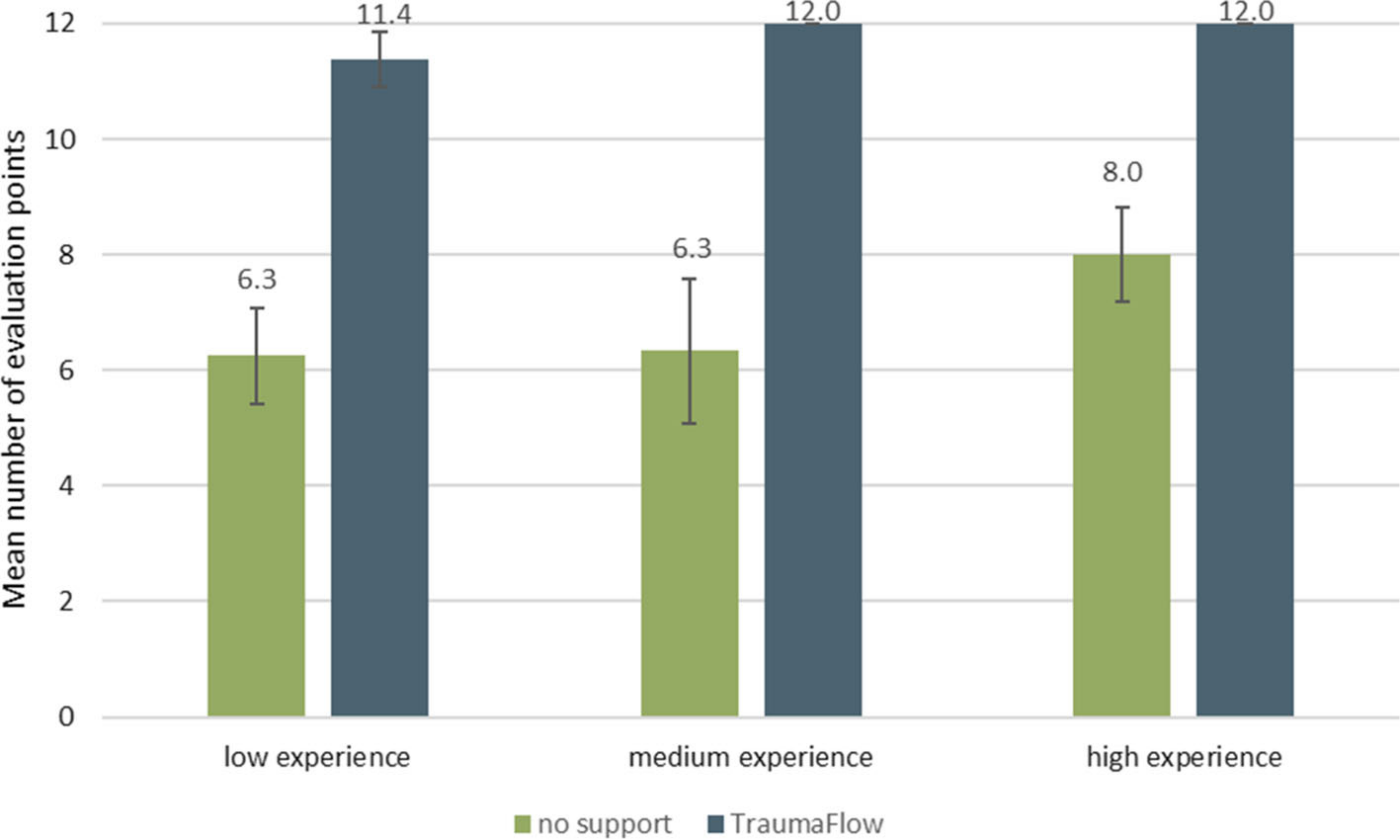
Number of evaluation points achieved by differently experienced participants

In Fig. 5, the number of evaluation points achieved by the differently experienced participants is shown. Participants with low and medium experience benefit most from the decision support (mean improvement for low experience: 5.1 points and medium experience: 5.7 points). Medium- and highly experienced participants performed perfect treatment with decision support. In total, a significant improvement in guideline adherence and completeness of treatment was achieved with the use of TraumaFlow (6.6 ± 1.2 points without vs. 11.6 ± 0.5 points with TraumaFlow, *p* < 0.00001 (Mann–Whitney-U-test)). A detailed analysis of the clinical evaluation can be found in [27].

#### System behavior

**Fig. 6:**
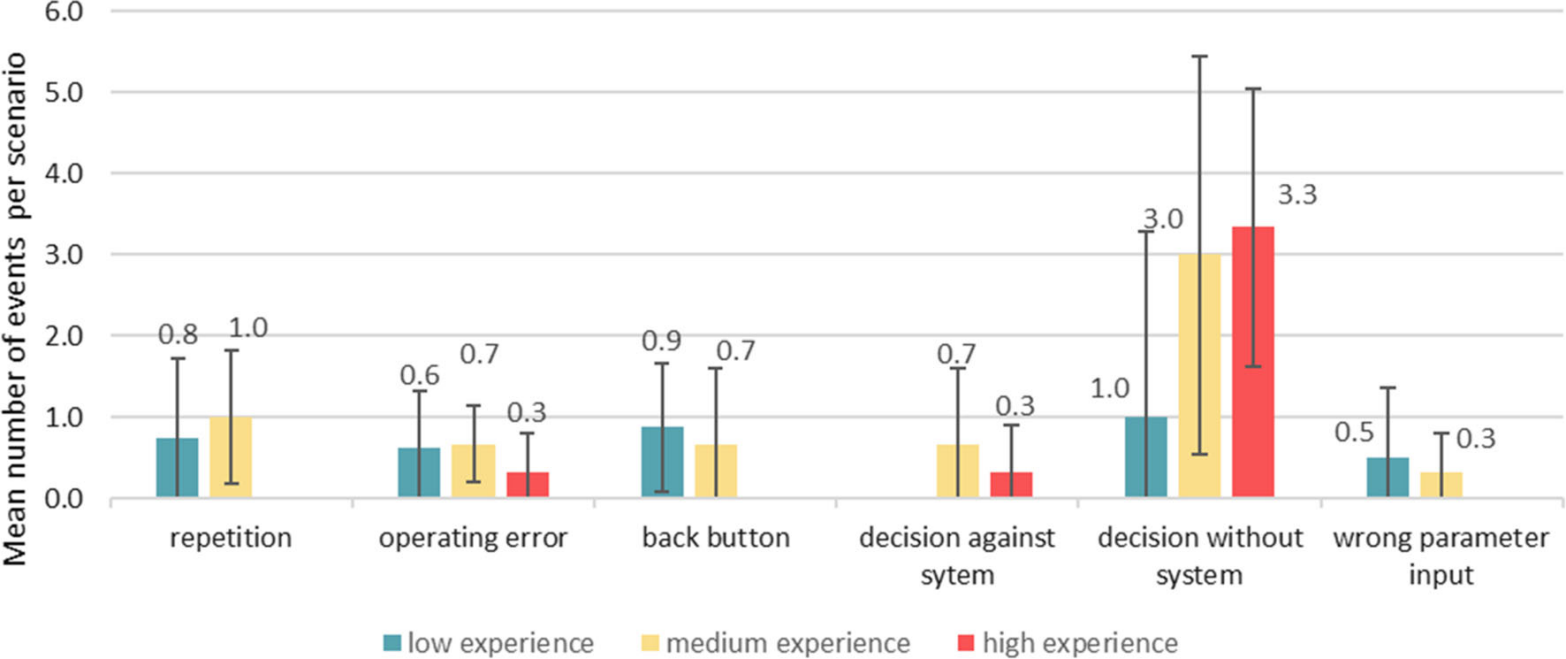
Usage errors of differently experienced participants

During scenario execution, the obstacles in the interaction of the participants with TraumaFlow were recorded. In Fig. 6, the mean number of usage errors per session is shown. ‘Unnecessary’ repetitions occurred due to contradictory parameter inputs, which is a security feature to reevaluate the user input. Repetitions were noted 0.6 ± 0.9 times per training scenario. Operating errors occurred due to ambiguous questions of the system 0.6 ± 0.6 times on average. To correct the user input, the ‘Back-Button’ was used 0.6 ± 0.8 times per scenario. In particular, active decisions against (0.2 ± 0.6 times per scenario) and without the recommendations of TraumaFlow (1.9 ± 2.5 times per scenario) were mostly made by medium- and highly experienced participants. Wrong parameter inputs that led to incorrect recommendations were noted in 0.4 ± 0.7 events per scenario.

**Fig. 7:**
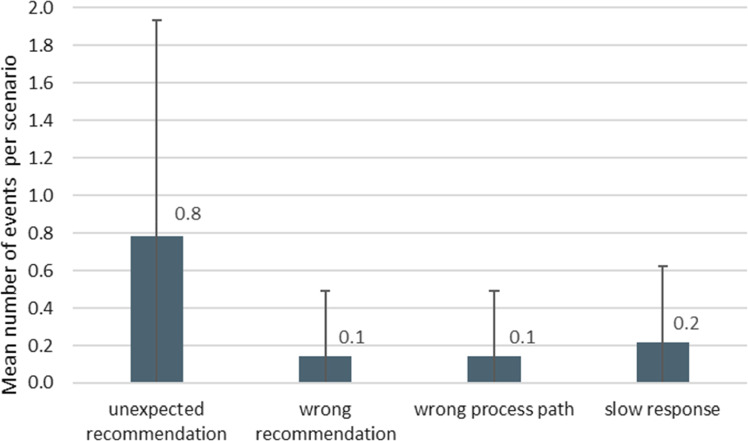
System errors during all training sessions

In addition, system errors such as user-unexpected recommendations, wrong recommendations, wrong process paths, and slow responses of the system were noted (Fig. 7). For the user, unexpected recommendations occurred 11 times in total. Thereof, two were wrong recommendations.

#### Human-system-interaction

**Fig. 8:**
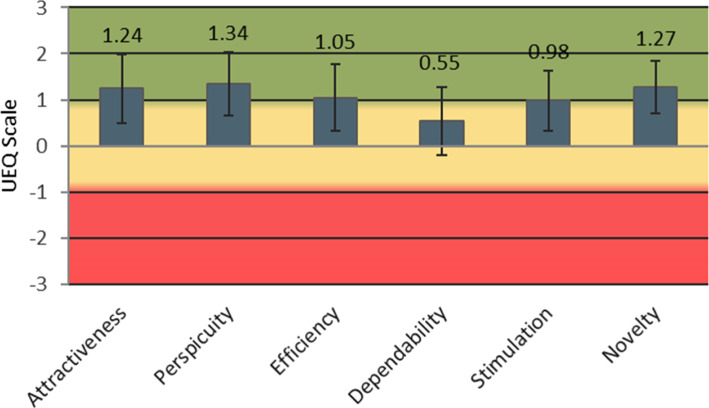
Results of the UEQ Questionnaire

The usability of TraumaFlow was evaluated by the participants with the standardized UEQ (User Experience Questionnaire) [28], which is used to assess the acceptance and users' subjective experience with software systems. The UEQ consists of 26 items related to six categories: Attractiveness (aesthetic and emotional impression), perspicuity (how easy to learn), efficiency (solving tasks without unnecessary efforts, fast usage), dependability (how secure and predictable), stimulation (how exciting and motivating), and novelty (how creative and innovative is the design). Users rate each item on a scale from − 3 to 3. Positive values over one represent a positive evaluation. TraumaFlow was evaluated positively in 5 of 6 categories (Fig. 8). The dependability was rated neutral due to slow system performance during several training sessions and user-unexpected recommendations.

**Fig. 9:**
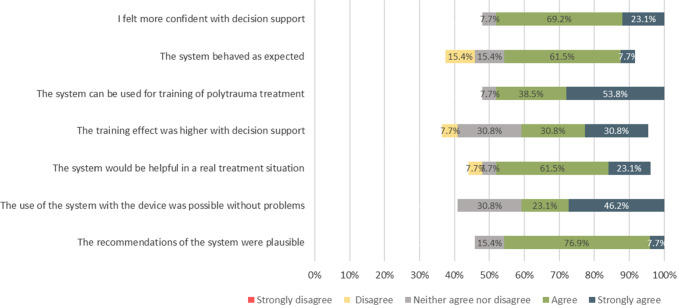
Results of the Likert Scale questions about user experience with TraumaFlow

In addition, the participants were asked to answer seven questions about the user experience with TraumaFlow (Fig. 9). The large majority (92%) of the participants felt more confident with computer-aided decision support and considered TraumaFlow useful for the training of polytrauma treatment (92%). In addition, 62% rated the training effect with support higher than without, and 85% believe that TraumaFlow can be helpful in a real treatment situation. Most participants (69%) could use the system with the tablet device without problems, and 85% considered the recommendations of the system plausible. While, 69% think the system behaves as expected, 15% experienced unexpected behavior.

**Fig. 10:**
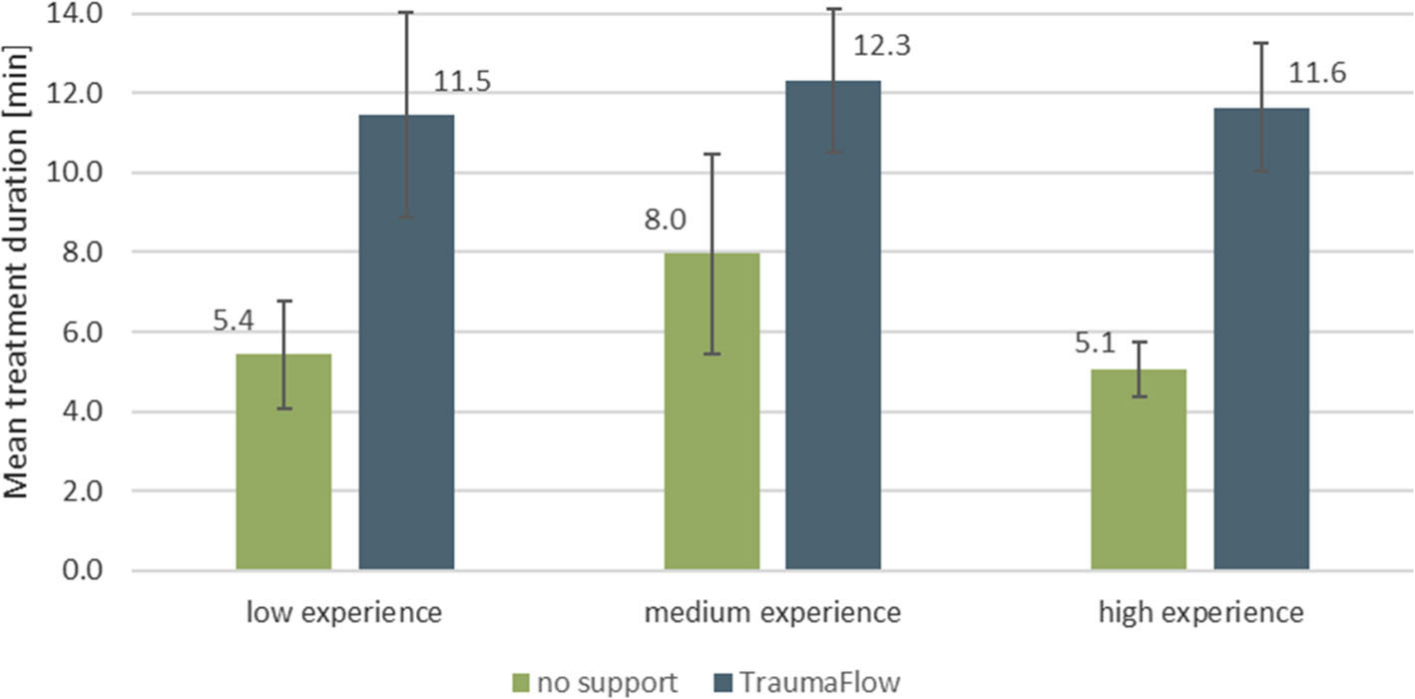
Treatment duration with and without TraumaFlow

For all training sessions, the treatment duration was recorded (Fig. 10). The participants were not given a time limit during the training, but the vital signs of the mannequin responded to incorrect or delayed treatment steps. Without TraumaFlow, the treatment was significantly shorter than with the system (mean time without: 5.9 min ± 1.9 min versus 11.7 min ± 2.3 min with TraumaFlow, *p* < 0.0001 (Mann–Whitney-U-Test)). The initial treatment in the real-world resuscitation room usually takes approximately 20–30 min until the patient is transferred to the operating room or intensive care.

## Discussion

In literature, CDSS are linked to a decrease in missed care steps, a reduction of treatment errors, and an increase in correctly performed treatment steps [18]. The results of the TraumaFlow evaluation study support these findings. Furthermore, the participants acknowledged that the system is helpful for polytrauma training and that the training effect was higher with the system. In a previous study, it was also shown that TraumaFlow significantly reduced self-reported mental stress during polytrauma training without increasing the overall workload [27].

With TraumaFlow it is possible to document the training session in detail and automatically generate training protocols, which improve the training effect, objectify the training evaluation, and increase comparability with previous training sessions. TraumaFlow was mainly developed to support the education of medical students. However, even highly experienced physicians achieved only 8.0 evaluation points on average (mean improvement: 4.0 with TraumaFlow), which indicates the complexity of the treatment algorithms. These findings suggest that experienced trauma leaders would also benefit from repeated simulation training to revive acquired knowledge and adherence to the latest treatment guidelines. Those guidelines are subject to continuous improvement due to new findings, emerging research, and changes in healthcare standards to enhance effective and safe patient care. There is a crucial need to ensure that treatment remains current with guidelines and that the latest improvements are implemented directly into medical education and training. Due to the model-based approach of TraumaFlow, the system can be easily updated to the latest guideline recommendations but is also exchangeable between healthcare institutions and adaptable to local conditions and preferences of the trauma team.

Recent advances in machine learning (ML) technologies have resulted in the rapid expansion of CDSS for diagnosis and treatment. Currently, these systems have difficulties providing a substantiated explainability of the decision-making process (xAI methods) [29], especially in real-time and specific to the actual situation. In addition, the training of the ML models may not be adaptive and fast enough to reflect the rapidly emerging best evidence. In contrast, TraumaFlow enables explainable, real-time decision support even for complex decisions by providing traceable workflow- and rule-based decision models. However, an ML component may improve user-adaptiveness in a future TraumaFlow version.

During the training session, some participants expressed dissatisfaction with the prolonged treatment time. The cases managed with the support of TraumaFlow required approximately six minutes more time than those managed without support. It remains unclear what effect a slightly prolonged treatment time has on patient outcome. In Germany, the time from hospital admission to the beginning of the CT scan is approximately 25 min [30]. In view of this timeline, six minutes seem a reasonable investment considering the improved guideline adherence and avoidance of potentially life-threatening errors. Furthermore, it can be assumed that as with all new technologies, with training and repetitive application in the clinical context, the additional time required will diminish over time [31].

In particular, the manual input of patient and medical device parameters caused frustration and stress for some participants [27]. In a future software version, an interface for vital sign monitoring will be developed to reduce manual input and accelerate the process. In addition, the system shall recognize potential uncertainties and adapt the level of decision support and explanations.

## Conclusion

In this work, TraumaFlow a workflow-based CDSS, was developed that enables patient-specific and real-time decision support for the treatment of polytrauma patients. A detailed cABCDE treatment algorithm was modeled with BPMN, and the workflow management system Flowable was instantiated. TraumaFlow supports the education of medical students but has also been shown to be beneficial for the training of highly experienced users. During an evaluation study, TraumaFlow improved guideline-based decision-making in complex and critical situations, provided a more complete treatment, and reduced human errors. Supporting functions, such as the automatic treatment documentation and the calculation of medical scores, enable the trainees to focus on the learning task. Each training session is documented in detail and can be objectively and qualitatively evaluated to improve learning.

## Data Availability

The datasets used and analyzed during the current study are available from the corresponding author on reasonable request.
